# Differential co-expression analysis reveals early stage transcriptomic decoupling in alzheimer’s disease

**DOI:** 10.1186/s12920-020-0689-y

**Published:** 2020-04-03

**Authors:** Yurika Upadhyaya, Linhui Xie, Paul Salama, Sha Cao, Kwangsik Nho, Andrew J. Saykin, Jingwen Yan, for the Alzheimer’s Disease Neuroimaging Initiative

**Affiliations:** 1 Department of BioHealth Informatics, Indiana University Purdue University Indianapolis, Indianapolis, IN USA; 2 Department of Electric and Computer Engineering, Indiana University Purdue University Indianapolis, Indianapolis, IN USA; 3 Department of Biostatistics, Indiana University School of Medicine, Indianapolis, IN USA; 4 Department of Radiology and Imaging Sciences, Indiana University School of Medicine, Indianapolis, IN USA

**Keywords:** Gene co-expression network, Stage-specific co-expression changes, Alzheimer’s disease

## Abstract

**Background:**

Alzheimer’s disease (AD) is one of the leading causes of death in the US and there is no validated drugs to stop, slow or prevent AD. Despite tremendous effort on biomarker discovery, existing findings are mostly individual biomarkers and provide limited insights into the transcriptomic decoupling underlying AD. We propose to explore the gene co-expression patterns in multiple AD stages, including cognitively normal (CN), early mild cognitive impairment (EMCI), late MCI and AD.

**Methods:**

We modified traiditonal joint graphical lasso to model our asusmption that the co-expression networks in consecutive disease stages are largely similar with critical differences. In addition, we performed subsequent network comparison analysis for identification of stage specific transcriptomic decoupling. We focused our analysis on top AD-enriched pathways.

**Results:**

We observed that 419 edges in CN, 420 edges in EMCI, 381 edges in LMCI and 250 edges in AD were frequently estimated with non zero weights. With modified JGL, the weight of all estimated edges in CN, EMCI and LMCI are zero. In AD group, 299 edges were occasionally estimated to be nonzero and the average correlation between genes was 0.0023. For co-expression change during AD progression, there are 66 pairs of genes that demonstrated a continuously decreasing or increasing co-expression from CN to EMCI, LMCI and AD.The network level clustering coefficient remains stable from CN to LMCI and then decreases significantly when progressing to AD. When evaluating edge level differences, we identified eight gene modules with continuously decreasing or increasing co-expression patterns during AD progression. Five of them shows significant changes from CN to EMCI and thus have the potential to serve system biomarkers for early screening of AD.

**Conclusion:**

We employed a modified joint graphical lasso for estimation of co-expression networks for multiple stages of AD. Comparing with graphical lasso, our modified joint graphical lasso model accounts for the similarity in consecutive disease stages. Our results on real data set revealed five gene clusters with obvious co-expression pattern change from CN to EMCI, which could be used as potential system-level biomarkers for early screening of AD.

## Background

Alzheimer’s disease (AD) is a major neurodegenerative disorder that has been characterized by gradual memory loss and brain behavior impairment. According to the latest report [1], an estimated number of 5.7 million aging Americans are living with Alzheimer’s and this number is expected to escalate in coming years given the rapid increase of aging population. To prevent this public health crisis, tremendous effort has been dedicated to discovery of effective AD biomarkers. In addition to *APOE* e4 alleles known as major genetic determinants [2], large-scale genome-wide association studies (GWAS) have led to identification of many novel genetic risk locus [3]. However, extant work largely investigated genetic variations or individual genes associated with AD. Very few studies paid attention to the interactions and associations among the gene products and how they are gradually disrupted during AD progression [4, 5].

Gene co-expression networks describes the correlation patterns among genes. Differential co-expression analysis examines the altered patterns between co-expression networks of two states, e.g., healthy controls vs. patients. It has great potential to identify the gene clusters affected by stage transition and therefore provide valuable information on how the biological system alters during disease progression. According to the Alzheimer’s Disease Neuroimaging Initiative (ADNI) project, subjects usually progress from cognitive normal (CN) status (i.e., those without any signs of depression, dementia or cognitive impairment), to early mild cognitive impairment (EMCI), late mild cognitive impairment (LMCI) (i.e., those with deteriorating memory concerns, but not decline in performing daily activities and no signs of dementia), and finally AD. In this project, we propose to perform a differential co-expression analysis across all the stages of AD, including CN, EMCI, LMCI and AD.

One common method to generate co-expression network is through pairwise Pearson’s correlation and WGCNA is the widely used package for co-expression analysis [6], which is based on marginal correlation. Although frequently used, these methods do not distinguish direct relationships from indirect relationships, e.g., two genes that show similar co-expression patterns due to a common intermediate gene. Unlike these methods, graphical lasso models the joint distribution of genes and can infer the direct relationships by capturing the conditional independence between genes [7]. A graph generated from graphical lasso, where genes are represented as nodes and their co-expression are represented as edges, provides valuable insights into the transcriptomic coupling under specific condition and therefore can help with generating new biological hypothesis. However, it can only estimate one network at a time. When applied to differential co-expression analysis, they estimate the network for each group separately by treating them as independent. This assumption clearly does not hold in disease research since disease formation is a progressive procedure. Co-expression networks in consecutive disease stages should be largely similar with critical differences. For example, co-expression networks in CN group and EMCI group are expected to be largely similar with critical differences. Toward this, we propose to employ joint graphical Lasso [8] for simultaneous estimation of co-expression networks in multiple disease stages. A joint analysis borrows the strength of the relatedness across disease stages and can potentially reveal the differential co-expression patterns with increased statistical power, which is useful especially when the sample size is very limited.

Given the increasing interest in and evidence of blood-based AD biomarkers [9–12], we focused our analysis on the plasma expression data of genes in top AD-enriched pathways highlighted in [13]. We first estimated the co-expression networks of all diagnosis groups using our modified JGL to better model our assumption of network similarity between consecutive disease stages. Subsequently, we performed a comprehensive network comparison analysis of co-expression networks using edge-level, node-level and network-level metrics. We used global clustering coefficient to identify structural property of each network. Node and edge centrality values were calculated to allow comparison of basic network components present in the network [14] and identification of critical network entities [15]. Finally, we were able to identify eight gene clusters showing gradual changes during the progression of AD. Five of them shows significant changes from CN to EMCI and therefore have the potential to serve as system level biomarkers for early screening of AD.

## Methods

### Dataset

All the plasma microarray data used in this study were directly obtained from the Alzheimer’s Disease Neuroimaging Initiative (ADNI) project (http://adni.loni.usc.edu). Raw expression values obtained directly from CEL files were pre-processed using the Robust Multi-chip Average (RMA) normalization method. The RMA normalized expression array data further went through several quality control (QC) steps [16]. First, we checked the sex of samples using sex-specific gene expression data, including XIST and USP9Y. Second, sample identity was verified on the basis of expression profiling to Omni2.5M genotype match using a Bayesian method to predict individual SNP genotypes from gene expression data. Detailed data description and the preprocessing steps can be found in ADNI LONI website (http://adni.loni.usc.edu). We focused our analysis on five AD-enriched pathways highlighted in [13]. For each pathway, we extracted all the involved genes from Metacore. In total, there are 75 genes included in this study (Table 1). In the microarray data, if there are multiple probes corresponding to the same gene, we choose the probe with the maximum mean expression to represent the gene. Gene expression values were adjusted for RNA integrity Number (RIN), baseline age and sex to remove potential bias. Finally, 662 ADNI 1 subjects without missing gene expression values were included. Shown in Table 2 is the detailed demographic information of all subjects. We will jointly learn the gene co-expression networks for four consecutive disease stages.

**Table 1:** 75 genes involved in AD enriched pathways

**Pathway**	**Genes**	**Total**
Immune response-Alternative complement pathway	*C3, CD46, CD55, CD59, CLU, CR1, ITGAM, ITGAX*	8
Development-Neurotrophin family signaling	*MAPK3, BAX, BRAF, GAB1, GRB2, HRAS, JUN, MAGED1, MAP2K1, MAP2K2, MAP3K1, MAPK1,MAPK8, PIK3R1, PIP5K1A, PTPN11, RAC1, RAF1, RAP1A, RAPGEF1, SHC1, SORT1, SOS1, TP53,AK1*	25
Neurophysiological process-NMDA-dependent postsynaptic long-term potentiation in CA1 hippocampal neurons	*AKT1, BRAF, CALM1, CREB1, EIF4E, GRB2, HRAS, MAP2K1, MAP2K2, MAPK1, MAPK3, MKNK1, PIK3R1, PRKACA, RAP1A, RAPGEF3, RPS6KA1, RPS6KB1, SHC1, XYLT*2	20
Cell adhesion-Ephrin signaling	*ADAM10, CDC42, EPHA1, EPHB1, FYN, GRB10, HRAS, KALRN, MAP3K7, MAP4K4, MAPK8, NCK1, NCK2, PAK1, PXN, RAC1, RAF1, RAP1A, RASA1, RHOA, SLA, SLC10A3, SRC, GRB2*	24
Neurophysiological process-nNOS signaling in neuronal synapses	*CALM1, CALM2, CALM3, DLG4, GRIN2C, GRINA, PPP3CA PPP3CB, PPP3CC, PPP3R1, PRKCB, PRKCD, PRKCH, PRKCI, PRKD3*	15

**Table 2:** Demographic information of participants

Groups	Total(N)	Gender (M/F)	Age (Mean ± Std)
CN	225	113/112	76.65 ±6.16
EMCI	193	105/88	79.26 ±7.35
LMCI	202	127/75	76.38 ±7.89
AD	42	27/15	75.69 ±9.46

### Joint graphical lasso

We first briefly introduce the graphical lasso method [7]. Suppose we have the expression data of *p* genes for *n* subjects in *K* diagnostic groups. Denote the gene expression profile for *k*-th group as $\mathbf {X}^{(k)} \subseteq \Re ^{n_{k}\times p}$, where *n*_*k*_ is the number of subjects in *k*-th group and $k=1,\dots,K$. Assuming that the gene expression levels within each group $\mathbf {x}_{1}^{(k)},x_{2}^{(k)},\cdots, x_{n}^{(k)}$ are independent and identically distributed with the positive definite *p*×*p* covariance matrix **Σ**_*k*_. The values in the covariance matrix reveal how the expression of two genes vary together; the inverse covariance matrix $\mathbf {\Sigma }_{k}^{-1}$ indicates the conditional independence between pairs of genes. Zero values in the inverse covariance matrix $\mathbf {\Sigma }_{k}^{-1}$ indicates conditionally independence of corresponding gene pairs. That is, two genes are independent of each other after removing the effect of all other genes in the data set.

Traditional graphical lasso estimates individual co-expression networks separately by solving Eq. 1:

1 $$ \underset{\mathbf{\Theta}^{(k)}}{\min} - \log\det\mathbf{\Theta}^{(k)}+trace\left(\mathbf{S}^{(k)}\mathbf{\Theta}^{(k)}\right) + \lambda_{1}||\mathbf{\Theta}^{(k)}||_{1})\\  $$

Where **S**^(*k*)^=(**X**^(*k*)^)^*T*^**X**^(*k*)^ is the empirical covariance matrix. ||**Θ**^(*k*)^||_1_ is the *L*_1_ norm. **Θ**^(*k*)^ is the inverse covariance matrix (i.e., co-expression network) to be estimated for *k*-th group. *λ*_1_ is the non-negative tuning parameter to enforce the sparsity of estimated co-expression network.

However, multiple groups may be related and ignoring the common structures shared across groups will inevitably yield sub-optimal results. To address this problem, joint graphical lasso (Eq. 2) was later proposed to enable the estimation of multiple related inverse covariance matrices together through maximizing penalized log likelihood [8]. It borrows the strength of the relatedness across groups and can potentially reveal the differential co-expression patterns with increased statistical power, which is especially useful when the sample size is very limited.

2 $$ {}\begin{array}{*{20}{c}} \underset{\{\mathbf{\Theta}\}}{\min} -\sum\limits_{k=1}^{K} n_{k} \left(\log\det\mathbf{\Theta}^{(k)}-trace\left(\mathbf{S}^{(k)}\mathbf{\Theta}^{(k)}\right)\right) + P(\{\mathbf{\Theta}\}) \\ \quad s.t. \quad \mathbf{\Theta }^{(1)},\dots,\mathbf{\Theta}^{(K)}>0 \\ \end{array}  $$

The sparsity within each covariance matrix and the similarity across covariance matrices in *K* groups are encouraged by penalty *P*({**Θ**}). *λ*_1_ and *λ*_2_ are two non-negative parameters to control the enforcement of sparsity and similarity. In [8], it assumed that the co-expression networks across all pair of groups are similar. However, this assumption does not always hold, especially for discovery of disease stage-specific networks. AD is a slowly progressive brain disorder that networks are expected to gradually dissolve or rewire during the progression. Gene co-expression network in the AD patients may have become very different compared to that of cognitive normals after years of progression. Therefore, we modified the penalty term *P*({**Θ**}) to Eq. 3 such that the similarity among networks is only enforced for consecutive stages. For example, networks between CN and EMCI and networks between EMCI and LMCI groups are encouraged to be similar.

3 $$ P\left({\left\{ { \boldsymbol{\Theta}} \right\}} \right) = {\lambda_{2}}\sum\limits_{k=1}^{K-1} {\sum\limits_{i \ne j} {\left| {\theta_{ij}^{\left(k \right)} - \theta_{ij}^{\left({k+1} \right)}} \right|} } + {\lambda_{1}}\sum\limits_{k = 1}^{K} {\sum\limits_{i \ne j} {\left| {\theta_{ij}^{\left(k \right)}} \right|} }  $$

To solve the modified JGL model, we followed the steps in [8] using alternating directions method of multipliers (ADMM) algorithm. With the constraints ***Θ***^(*k*)^=***Z***^(*k*)^, the dual variables ***U***^(*k*)^ are introduced to form scaled augmented Lagrangian [17].

4 $$\begin{array}{*{20}l} {}L\left({\left\{ \boldsymbol{\Theta} \right\}, \left\{ \boldsymbol{Z} \right\}, \left\{ \boldsymbol{U} \right\}} \right) = & -\! \sum\limits_{k = 1}^{K} {{n_{k}}\!\left({\log \det {\boldsymbol{\Theta }^{\left(k \right)}} \,-\, tr\left({{\boldsymbol{S}^{\left(k \right)}} {\boldsymbol{\Theta }^{\left(k \right)}}} \right)} \right)} \\ & + \frac{\rho }{2}\sum\limits_{k = 1}^{K} {\left\| {{\boldsymbol{\Theta }^{\left(k \right)}} - {\boldsymbol{Z}^{\left(k \right)}} + {\boldsymbol{U}^{\left(k \right)}}} \right\|}_{F}^{2}  \\ & + P\left({\left\{ \boldsymbol{Z} \right\}} \right) \end{array} $$

The exact solution steps can be referred to the JGL paper [8]. We modified the step to update {***Z***} (Eq. 5).

5 $$ \mathop { \text{min} }\limits_{\left\{ \boldsymbol{Z} \right\}} \left[ {\frac{\rho }{2} \sum\limits_{k = 1}^{K} {\left\| {\boldsymbol{Z}^{\left(k \right)} - \boldsymbol{A}^{\left(k \right)}} \right\|}_{F}^{2} + P\left(\left\{ \boldsymbol{Z} \right\} \right) } \right]  $$

We found that this minimization problem is completely separable for each element (i,j) in the matrices,

6 $$\begin{array}{*{20}l} \mathop { \text{min}}\limits_{Z_{ij}^{\left(1 \right)},...,Z_{ij}^{\left(K \right)}} \quad & \frac{\rho}{2} \sum\limits_{k = 1}^{K} {\left\| {Z_{ij}^{\left(k \right)} - A_{ij}^{\left(k \right)}} \right\|}_{F}^{2} + {\lambda_{1}}\sum\limits_{\scriptstyle k = 1\hfill\atop \scriptstyle i \ne j\hfill}^{K} {\left| {Z_{ij}^{\left(k \right)}} \right|}  \\ & + {\lambda_{2}}\sum\limits_{k=1}^{K-1} \left| {Z_{ij}^{\left(k \right)} - Z_{ij}^{\left({k + 1} \right)}} \right| \end{array} $$

The last penalty term is known as 1-*d* fused lasso and penalizes the absolute differences in adjacent values of **β** = $[ \boldsymbol {Z}_{ij}^{(1)},\dots,\boldsymbol {Z}_{ij}^{(K)}]$. It can be easily reformulated as ∥**Dβ**∥_1_, where **D**⊆ℜ^(*K*−1)×*K*^.

7 $$ \mathbf{D} = \left[\begin{array}{llllll} -1 & 1 & 0 & \dots & 0 & 0 \\ 0 & -1 & 1 & \dots & 0 & 0 \\ & & & \dots & & \\ 0 & 0 & 0 & \dots & -1 & 1 \\ \end{array}\right]  $$

If we first consider *λ*_1_ as zero and set **y**=$\left [\mathbf {A}_{ij}^{(1)},\dots,\mathbf {A}_{ij}^{(K)}\right ]$. The convex optimization problem becomes a simple 1-*d* fused lasso problem (Eq. 8). Note that rank(**D**)= *K*−1. With a vector **s**⊆ℜ^*K*^ that is orthogonal to all the rows in **D**, we can construct $\hat {\mathbf {D}}=\left [\begin {array}{c}\mathbf {D}\\ \mathbf {s}\end {array}\right ]$ with rank($\hat {\mathbf {D}}$)=*K*. Let $\theta = (\theta _{1},\theta _{2})^{T} = \hat {\mathbf {D}}\beta $ and **θ**_1_⊆ℜ^*K*−1^, then Eq. 8 will be transformed into a regular lasso problem (Eq. 9).

8 $$ \underset{\{\mathbf{\beta}\}}{\min} \quad \frac{\rho}{2}||\mathbf{\beta}-\mathbf{y}||_{F}^{2} + \lambda_{2} \|\mathbf{D\beta}\|_{1}  $$

9 $$ \underset{\{\mathbf{\theta}\}}{\min} \quad \frac{\rho}{2}||\hat{\mathbf{D}}^{-1}\theta-\mathbf{y}||_{F}^{2} + \lambda_{2} \|\mathbf{\theta}_{1}\|_{1}  $$

Note that the L1 penalty is only partially applied to **θ**_1_. In order to solve this, we re-write Eq. 9 to a standard form with transformation $\hat {\mathbf {D}}^{-1}\theta = \left [ {\begin {array}{*{20}{c}} {{}^{K\backslash K-1}\boldsymbol {X}_{1}}&{\left | {} \right.{}^{K\backslash 1}\boldsymbol {X}_{2}} \end {array}} \right ]{\left [ {\begin {array}{*{20}{c}} {\boldsymbol {\theta }_{1} {} }&{\left | {} \right.\boldsymbol {\theta }_{2}} \end {array}} \right ]^{T}}$,

10 $$ \mathop {{\text{min}}}\limits_{\boldsymbol{\theta}_{1}, \boldsymbol{\theta}_{2}} {\frac{\rho }{2}\left|| {\left({\boldsymbol{y} - \boldsymbol{X}_{1}\boldsymbol{\theta}_{1}} \right) - \boldsymbol{X}_{2}\boldsymbol{\theta}_{2}} \right||_{F}^{2} + {\lambda_{2}}\left|| {\boldsymbol{\theta}_{1}} \right||_{1}}  $$

While $\boldsymbol {\hat {\theta }_{2}}$ can be solved as $\boldsymbol {\hat {\theta }_{2}} = {\left ({\boldsymbol {X}_{2}^{T}{\boldsymbol {X}_{2}}} \right)^{- 1}} \boldsymbol {X}_{2}^{T}\left ({\mathbf {y} - {\boldsymbol {X}_{1}}{\boldsymbol {\hat \theta }_{1}}} \right)$, the above equation can be rewritten as

11 $$  \mathop {{\text{min}}}\limits_{\boldsymbol{\theta}_{1}} {\frac{\rho }{2}\left|| {\left({(\mathbf{I}-\mathbf{P})\mathbf{y} - (\mathbf{I}-\mathbf{P}) \boldsymbol{X}_{1}\boldsymbol{\theta}_{1}} \right)} \right||_{F}^{2} + {\lambda_{2}}\left|| {\boldsymbol{\theta}_{1}} \right||_{1}}  $$

Where $\boldsymbol {P}= \boldsymbol {X}_{2} {\left ({ \boldsymbol {X}_{2}^{T}\boldsymbol {X}_{2}} \right)^{- 1}} \boldsymbol {X}_{2}^{T}$. The above equation can be easily solved using the LARS algorithm [18], from which we can back-transform to get the solution for Eq. 8: $\beta = \hat {\mathbf {D}}^{-1}\theta $. By applying soft-thresholding to *β*, we can get the solution for Eq. 6.

### Tuning parameter selection

The tuning parameters *λ*_1_ and *λ*_2_ in the modified JGL model were selected using the Akaike information criterion (AIC) (Eq. 12).

12 $$ {}AIC(\lambda_{1},\!\lambda_{2}) \,=\,\! \sum\limits_{k = 1}^{K}\!\left[ n_{k} trace\left(\!\mathbf{S}^{(k)}\hat{\mathbf{\Theta}}_{\lambda_{1},\lambda_{2}}^{(k)}\!\right)\,-\,log det \hat{\mathbf{\Theta}}_{\lambda_{1},\lambda_{2}}^{(k)} \!\,+\, 2E_{k}\right]  $$

Here, $\hat {\mathbf {\Theta }}_{\lambda _{1},\lambda _{2}}^{(k)}$ is the estimated inverse covariance matrix for group *k* under the tuning parameters *λ*_1_ and *λ*_2_. *E*_*k*_ is the number of non-zero elements in $\hat {\mathbf {\Theta }}_{\lambda _{1},\lambda _{2}}^{(k)}$. We performed a grid search and *λ*_1_ and *λ*_2_ with minimal AIC score were selected.

### Performance evaluation

In the subsequent gene co-expression analysis, we refer our modified JGL model as JGL. To evaluate the performance of JGL, we compared the performance of joint graphical lasso and traditional graphical lasso, where the co-expression network of each group is estimated separately. Using 1000 permuted datasets, we generated 1000 co-expression networks using JGL and derived a frequency network for each group, where each link has a value between 0 and 1000 indicating how many times it is observed to be nonzero. Similarly, we generate another frequency network for each group using the results from graphical lasso. Since all the gene expression has been permuted and they should be independent from each other, the ground truth value for all edges should be zero. Any nonzero values will be considered as false positives. That is, the value of each edge in the frequency network indicates its chance to be a false positive. We compared the performance of JGL and graphical lasso in terms of how frequently we will observe the false positives using the permuted data set.

### Construction of co-expression network

We applied the modified JGL model to estimate the co-expression networks of four diagnostic groups simultaneously. To evaluate the significance of the estimated networks, we generated a random data set by running 1000 times of permutation for each gene and each group. Random data sets were then fed into the modified JGL model to estimate 1000×4 co-expression networks. For each group, we compared our results against these randomly generated networks and estimated an empirical p value for each edge in each group. Edges with empirical P >0.05 were considered insignificant. There were no insignificant edges identified for CN, EMCI and LMCI groups. Five edges in AD were found to be insignificant and were removed from subsequent network comparison analysis.

### Centrality of the network nodes

For every node in the network, four weighted centrality values were calculated: weighted degree, weighted betweeness, weighted closeness and weighted clustering coefficient. Weighted degree is the sum of number of connections in terms of the weight of each edge in the network [19]. R package tnet provides the platform to calculate weighted centrality of a network. The weighted degree method proposed method by [20] uses the tuning parameter alpha to tune the connections and connection weights. The alpha value of 1 was used to calculate weighted degree. When an alpha value is 1, it equals to the definition of weighted degree by [19]. That is, degree is based on the connection weights of the network [19, 21]. Similarly, betweenness and closeness centrality are based on the theory of shortest paths in a network. closeness implies to how close a node is to other nodes in a network. Betweenness is the measure of how often an edge lies in the path of the other edge. We used alpha value as 1 for both calculation. Finally, clustering coefficient explains how connected neighbours of a node are in an overall network. Arithmetic mean measure was used to calculate the global clustering coefficient of the network as well as nodal clustering coefficients [20].

### Network comparison

We examined and compared all four co-expression networks using edge level, node level and network level metrics. For each edge, its weight indicates the correlation between two connecting genes. We compared four networks by each edge and looked for edges with continuously increasing or decreasing weights from CN to EMCI, LMCI and AD. Edges with absolute differences >0.01 in consecutive groups (i.e, (CN- EMCI), (EMCI -LMCI) or (LMCI - AD)) were considered as potential biomarkers. We further clustered these edges based on their patterns of change across four disease stages. Similarly, we also compared the centrality values of each node and edge to identify nodes with gradual changes from CN to EMCI, LMCI and AD.

## Results

### Comparison of jGL and graphical lasso

**Fig. 1:**
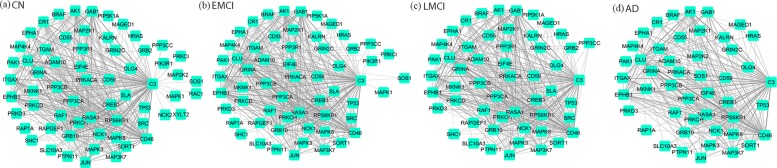
Frequency network generated using graphical lasso results on permuted data. Edges detected in more than 100 out of 1000 permuted data sets are plotted for **a** CN, **b** EMCI, **c** LMCI and **d** AD groups respectively

We examined the difference of our modified JGL and graphical lasso in terms of their performance in estimation of co-expression networks based on permuted data sets. While the permuted data sets are completely random, it is expected that none of the genes are correlated. So all the edges in the estimated networks should have zero weight. However, with networks estimated using graphical lasso, we observed that 419 edges in CN, 420 edges in EMCI, 381 edges in LMCI and 250 edges in AD were frequently (i.e., more than 100 out of 1000 times) estimated with non zero weights (i.e., false positives) (Fig. 1). With modified JGL, the weight of all estimated edges in CN, EMCI and LMCI are zero. In AD group, 299 edges were occasionally (i.e., less than 100 out of 1000 times) estimated to be nonzero and the average correlation between genes was 0.0023 (Additional file 1: Fig. S1).

### Nodal centrality change during aD progression

**Fig. 2:**
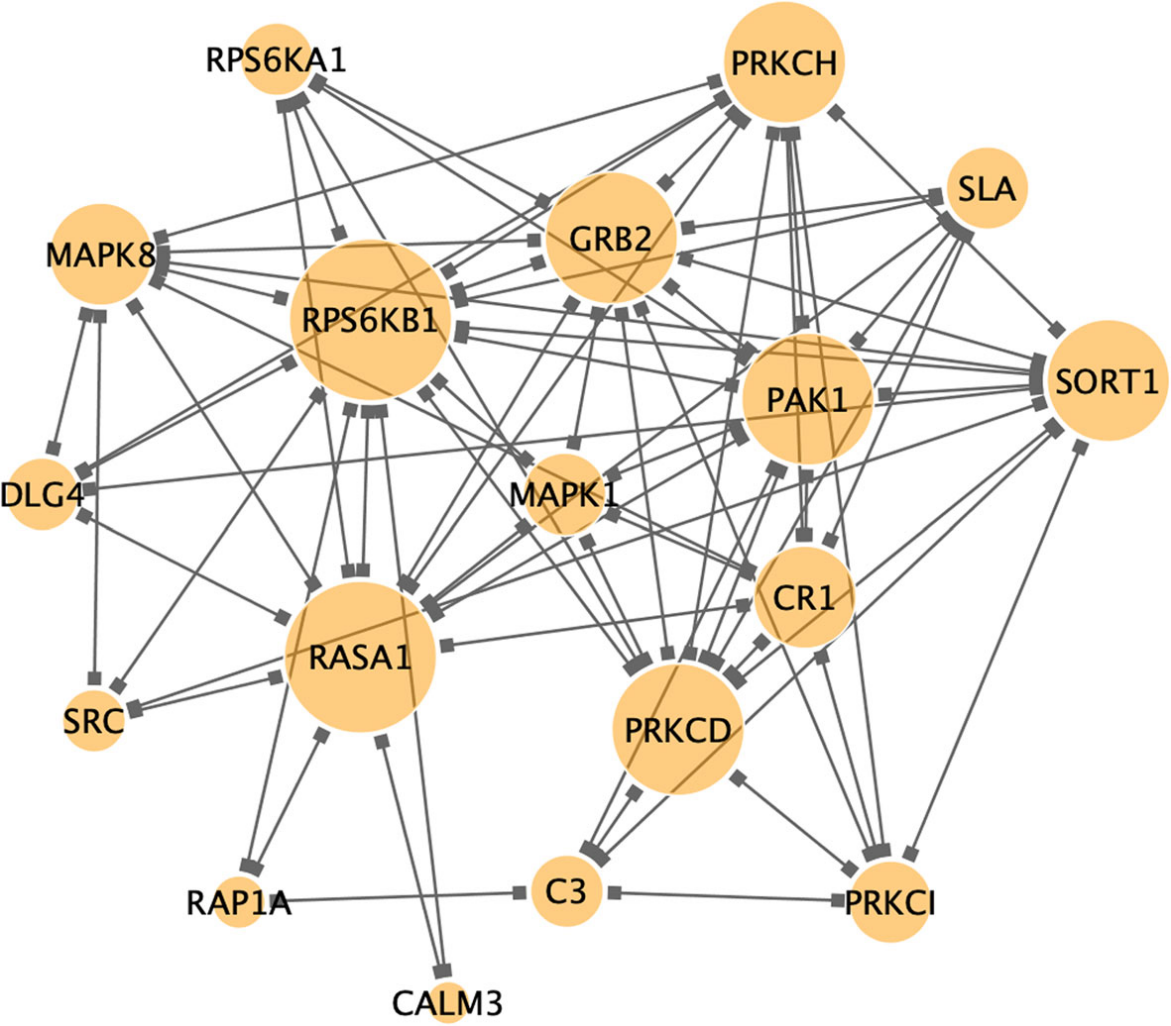
Network module with genes showing continuous nodal centrality change from CN to AD

There are totally 19 genes showing significant nodal centrality changes from CN to AD. For clustering coefficient, we found *C3*, *MAPK1*, *PAK1*, *PRKCH* and *SLA* with continuously increasing values from CN to EMCI, LMCI and AD. In contrast, *CALM3*, *MAPK8* and *RASA1* showed a decreasing pattern. For degree centrality, *MAPK8* and *RHOA* were found to increase and *GRB2*, *PAK1*, *PRKCD*, *PRKCH*, *PRKCI*, *SORT1* were found to decrease when subjects progress to a more severe stage. For betweenness, *CR1*, *GRB2* and *RPS6KB1* demonstrated a decreasing pattern. For closeness, *DLG4* and *SRC* were observed to increase while *PRKCD*, *RAP1A* and *RPS6KA1* showed a decreasing pattern. Among these, early centrality changes are captured by *RPS6KB1*, *MAPK8*, *PRKCD*, *PRKCH* and *CR1* from CN to EMCI. A complete list of genes with continuous centrality changes is shown in Table 3. The detail centrality values of identified genes are in (Additional file 1: Tables S2–S5.) In Fig. 2 is the co-expression network module in the CN group that includes only genes showing gradual nodal changes. *RPS6KB1* is observed to be the top hub.

**Table 3:** List of genes with continuous nodal centrality change

**Centrality**	**Type**	**Nodes**
	I	C3, MAPK1, PAK1, PRKCH, SLA
ClusCoef	D	CALM3, MAPK8, RASA1
	I	MAPK8, RHOA
Degree	D	GRB2, PAK1, PRKCD, PRKCH, PRKCI, SORT1
	I	None
Betweeness	D	CR1, GRB2, RPS6KB1
	I	DLG4, SRC
Closeness	D	PRKCD, RAP1A, RPS6KA1

ClusCoef: clustering coefficient; I: increasing; D: decreasing.

### Co-expression change during aD progression

**Fig. 3:**
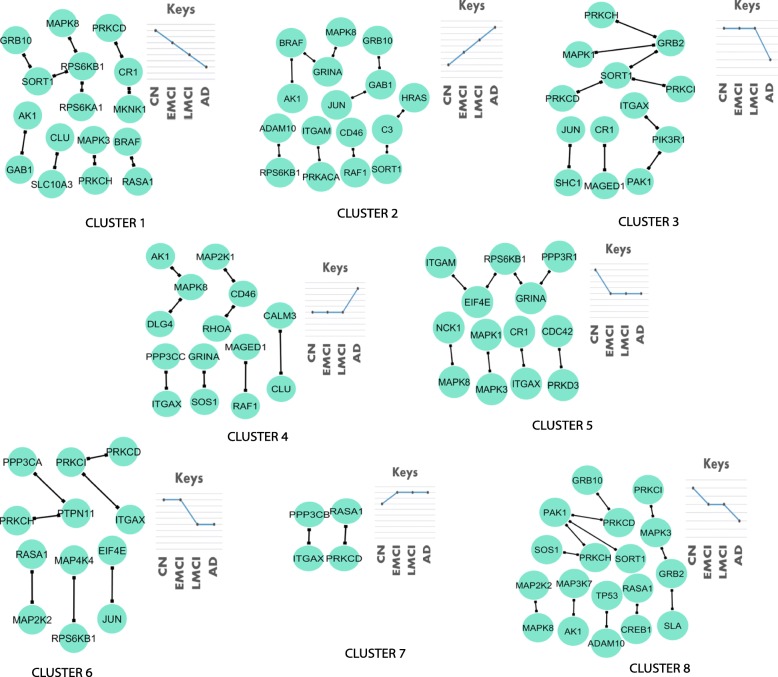
Eight gene clusters with distinct patterns of gene co-expression change from CN to AD

**Fig. 4:**
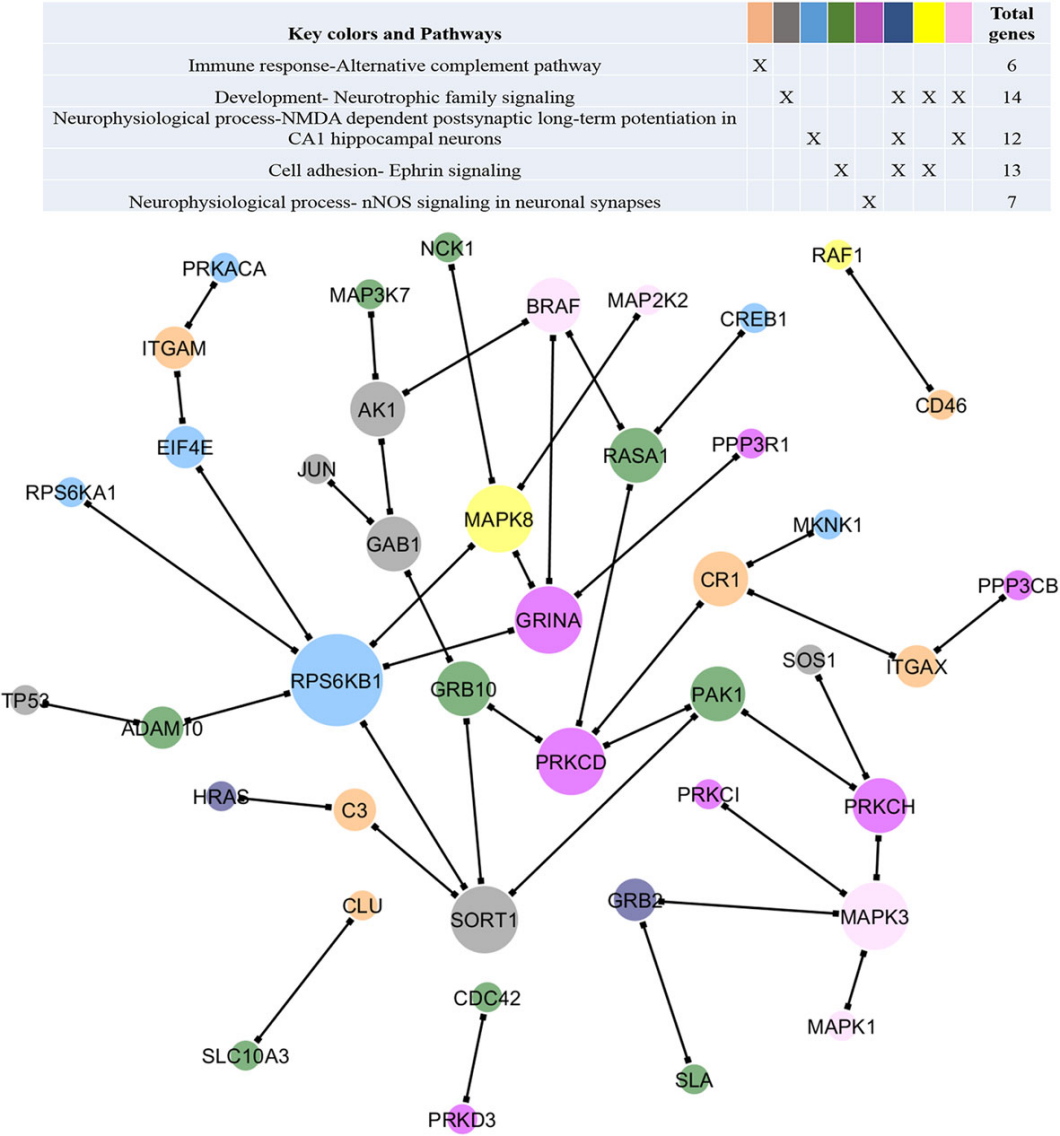
Network module where gene co-expression (i.e.,links) exhibit early change from CN to EMCI. Node color indicates their membership in different pathways

There are 66 pairs of genes that demonstrated a continuously decreasing or increasing co-expression from CN to EMCI, LMCI and AD. Based on their change patterns, we further divided these genes into eight clusters (Fig. 3). For clusters 1 and 2, correlation between those gene pairs shows continuous increase or decrease from CN to AD. For clusters 3 and 4, correlation between those gene pairs remains relatively stable until LMCI. On the contrary, gene pairs in clusters 5 and 7 show significant correlation change from CN to EMCI, but then remain stable until AD. After merging all of these modules, we observed several hub genes including *RPS6KB1* (Ribosomal Protein S6 Kinase B1), *SORT1*, *MAPK3*, textitPRKCD, *MAPK8* and *GRINA*. We further performed an pathway enrichment analysis for these clusters using hyper-geometric test (Additional file 1: Table S1). Five candidate pathways are significantly enriched for all clusters except cluster 7 and 8. Among eight gene clusters, early correlation changes occurred in five of them, either decreasing or increasing. We combined these five gene clusters and formed a gene module as shown in Fig. 4. *RPS6KB1* (Ribosomal Protein S6 Kinase B1) was found to be the hub gene, followed by *GRINA*, *MAPK3*, *PRKCD*, *MAPK8* and *SORT1*.

### Global network change during aD progression

**Fig. 5:**
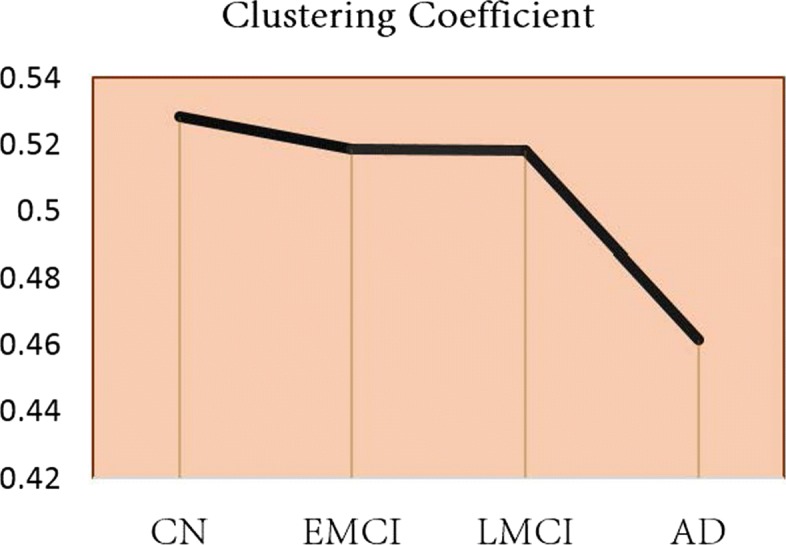
Global weighted clustering co-efficient for co-expression networks derived from our JGL model

Shown in Fig. 5 is the global clustering coefficient change of co-expression networks from CN to AD. It shows that the overall weighted clustering coefficient of the co-expression network remains relatively stable from CN to EMCI.

## Discussion

The superior performance of modified JGL over traditional graphical lasso, when using the randomly permuted data, suggests that the similarity constraints on the co-expression networks of consecutive disease stages can help effectively control the probability to generate false positives. Therefore, the differential co-expression patterns identified through JGL results will provide more accurate information of the altered biological system during disease formation. On the other hand, while the high false positive rate of traditional graphical lasso could be controlled by the multiple test correction, it will be subject to the selection of correction method and significance threshold. In contrast, the similarity constraint used in JGL played a key role in controlling the false positive rate even without multiple correction. Therefore, JGL does not require as large number of permutations as traditional JGL and is more computationally cost-effective.

The global clustering coefficient change of co-expression networks from CN to AD suggests strong structural resilience of the co-expression network in the early development of AD. However, the global clustering coefficient drops significantly when patients progress from LMCI to AD, which indicates the overall decoupling of genes in AD-enriched pathways starts dissolve at the late stage of AD. There is less probability to hold modular structures within co-expression network when the disease progresses from less severe to more severe stages.

Among all the genes showing significant changes in node or edge level metrics, *RPS6KB1* has consistently been observed as the top hub. Given that its centrality and co-expression changes occur in the early stage of AD, it holds great potential to serve as the biomarker for early diagnosis of AD. *RPS6KB1* encodes for a Ser/Thr (S/T)-directed kinase, 70-kDa S6 kinase (p70S6K), that plays a crucial role in cell growth, cell differentiation, and cell cycle control. The p70S6K can phosphorylate tau at S262, S214, and T212 sites [22]. It is found to be highly expressed in cerebral cortex and hippocampus, a brain region affected by AD, according to the Human Protein Atlas (HPA) [23]. In a large-scale genome wide association study (GWAS), it was reported to be a genetic risk loci for multiple sclerosis [24]. Findings from earlier studies suggest that the additive effect of alleles in *RPS6KB1* and several other genes in tau kinase pathway are associated with late-onset of AD in *APOE* non-e4 carriers [22, 25].

*MAPK8*, also known as *JNK1*, is one of the three proteins in c-Jun N-terminal Kinase family. It is identified to be involved in the regulation and maintenance of physiological responses in central nervous system [26]. Studies have also found positive correlation between JNK phosphorylation and expression of amyloid beta (A *β*) peptide, which is a well-known AD hallmark[27].

Other top hub genes that also show early changes in nodal centrality or co-expression patterns are *MAPK8*,*MAPK3*, *GRINA*, *PRKCD*, *SORT1*, *RASA1* and *PAK1*. A few studies have reported the association of these genes with AD. For example, [28] suggests a potential causative role of P21 activated kinases defects on the cognitive deficits of AD patients. However, their coordination has not been previously studied yet. This work is the first study to reveal how their co-expression pattern changes during AD progression. Given that the co-expression and nodal centrality of these genes start to change in the CN stage, they have great potential to serve as systems biomarker to capture the biological alterations in the very early stage of AD and further help with the development of AD therapeutic intervention.

## Conclusion

We employed a modified joint graphical lasso for estimation of co-expression networks for multiple stages of AD. Comparing with graphical lasso, JGL accounts for the similarity in consecutive disease stages. Our results on random data sets shows that it is less likely to generate false positives. In the subsequent differential co-expression analysis, we found that the clustering coefficient of the co-expression network shows significant changes only when subjects progress from LMCI to AD. Node wise and edge wise comparison have led to eight gene clusters that demonstrate continuous changes from CN, EMCI to LMCI and AD. Particularly, five of them shows differential co-expression patterns from CN to EMCI. Genes in these modules could be used as systems biomarkers for early screening in AD. However, more efforts are warranted to validate their downstream function.

## Supplementary information

**Additional file 1** Differential co-expression analysis reveals early stage transcriptomic decoupling in Alzheimer’s disease.

## Data Availability

Demographic information, gene expression data, and diagnostic information are available from the ADNI data repository at http://adni.loni.usc.edu/data-samples/access-data/.

## Abbreviations

A *β*: Amyloid *β*-protein
AD: Alzheimer’s disease
ADNI: Alzheimer’s disease neuroimaging initiative
AIC: Akaike information criterion
CN: Cognitive normal
EMCI: Early mild cognitive impairment
GWAS: Genome-wide association studies
JGL: Joint graphic Lasso
Lasso: Least absolute shrinkage and selection operator
LMCI: Late mild cognitive impairment
LONI: Laboratory of neuro imaging
QC: Quality control
RMA: Robust multi-chip average
SNP: Single Nucleotide Polymorphism
WGCNA: Weighted correlation network analysis
