# Supplementary material for: Differential co-expression analysis reveals early stage transcriptomic decoupling in alzheimer’s disease
Source: BMC Med Genomics. 2020 Apr 3;13(Suppl 5):53. doi: 10.1186/s12920-020-0689-y (PMC7118822; doi:10.1186/s12920-020-0689-y)

Supplementary Document:

Differential co-expression analysis reveals early stage transcriptomic decoupling in Alzheimer's disease

Supplementary Tables:

Supplementary Table 1: Pathway enrichment analysis results for 8 gene clusters. Shown in the table are the p-values derived from hypergeometric test.

Pathway	Cluster 1	Cluster 2	Cluster 3	Cluster 4	Cluster 5	Cluster 6	Cluster 7	Cluster 8
Immune response - Alternative complement pathway	1.7e-5	2.3e-8	1.1e-5	1.5e-8	1.2e-8	4.8e-3	1.6e-3	1.0
Development-Neurotrophic family signaling	1.6e-14	0.0	0.0	6.0e-15	4.9e-7	3.8e-7	1.0	0.0
Neurophysiological process- NMDA-dependent postsynaptic long-term potentiation in CA1 hippocampal neurons	2.5e-12	1.3e-9	5.2e-10	1.4e-2	5.2e-10	1.9e-7	1.0	2.2e-9
Cell adhesion- Ephrin signaling	2.9e-9	6.9e-12	1.1e-4	5.5e-7	4.3e-7	9.0e-5	4.8e-3	0.0
Neurophysiological process -nNOS signaling in neuronal synapses	6.3e-5	1.2e-2	9.7e-8	2.0e-10	9.7e-8	1.0e-10	3.1e-6	2.8e-7

Supplementary Table 2: Clustering coefficient of nodes showing continuous change from CN to AD.

Type	Gene	CN	EMCI	LMCI	AD
Increasing	C3	0.011	0.012	0.013	0.014
	MAPK1	0.039	0.040	0.040	0.051
	PAK1	0.029	0.030	0.033	0.037
	PRKCH	0.012	0.012	0.015	0.042
	SLA	0.034	0.038	0.039	0.041
Decreasing	CALM3	0.064	0.055	0.050	0.049
	MAPK8	0.043	0.034	0.031	0.020
	RASA1	0.020	0.020	0.019	0.017

Supplementary Table 3: Weighted degree of nodes showing continuous change from CN to AD.

Type	Gene	CN	EMCI	LMCI	AD
Increasing	MAPK8	0.885	0.971	1.017	1.201
	RHOA	0.014	0.014	0.019	0.037
Decreasing	GRB2	0.808	0.745	0.738	0.585
	PAK1	1.067	1.044	0.884	0.833
	PRKCD	1.180	0.977	0.965	0.751
	PRKCH	1.139	0.747	0.703	0.672

	PRKCI	0.382	0.378	0.204	0.130
	SORT1	1.089	1.053	0.894	0.848

Supplementary Table 4: Betweenness of nodes showing continuous change from CN to AD.

Type	Gene	CN	EMCI	LMCI	AD
Decreasing	CR1	288	199	173	125
	GRB2	26	21	18	4
	RPS6KB1	793	642	571	371

Supplementary Table 5: Closeness of nodes showing continuous change from CN to AD.

Type	Gene	CN	EMCI	LMCI	AD
Increasing	DLG4	64.012	71.266	72.751	76.285
	SRC	48.548	55.109	55.535	71.405
Decreasing	PRKCD	80.237	71.528	67.387	60.219
	RAP1A	83.124	82.194	81.122	78.073
	RPS6KA1	55.017	53.578	49.255	40.542

Supplementary Figure:

Supplementary Figure 1: Distribution of average pairwise gene correlation derived from the modified JGL and random data in AD group.

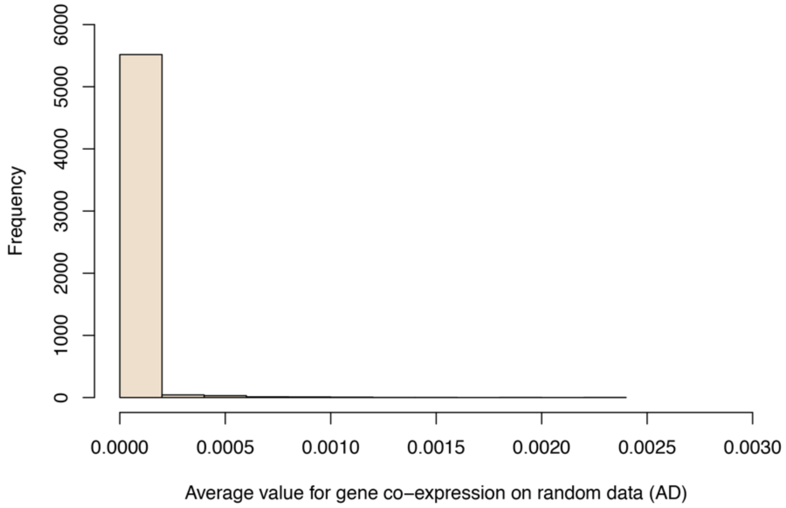

Supplement: Supplementary file 1 — Additional file 1 Differential co-expression analysis reveals early stage transcriptomic decoupling in Alzheimer’s disease. [file 12920_2020_689_MOESM1_ESM.pdf]
